# Salicylic acid reverses pollen abortion of rice caused by heat stress

**DOI:** 10.1186/s12870-018-1472-5

**Published:** 2018-10-19

**Authors:** Baohua Feng, Caixia Zhang, Tingting Chen, Xiufu Zhang, Longxing Tao, Guanfu Fu

**Affiliations:** State Key Laboratory of Rice Biology, China National Rice Research Institute, 359 Tiyuchang Road, Hangzhou, 310006 People’s Republic of China

**Keywords:** *Oryza sativa* L., Heat stress, Salicylic acid, H_2_O_2_, Pollen viability, Tapetum

## Abstract

**Background:**

Extremely high temperatures are becoming an increasingly severe threat to crop yields. It is well documented that salicylic acid (SA) can enhance the stress tolerance of plants; however, its effect on the reproductive organs of rice plants has not been described before. To investigate the mechanism underlying the SA-mediated alleviation of the heat stress damage to rice pollen viability, a susceptible cultivar (Changyou1) was treated with SA at the pollen mother cell (PMC) meiosis stage and then subjected to heat stress of 40 °C for 10 d until 1d before flowering.

**Results:**

Under control conditions, no significant difference was found in pollen viability and seed-setting rate in SA treatments. However, under heat stress conditions, SA decreased the accumulation of reactive oxygen species (ROS) in anthers to prevent tapetum programmed cell death (PCD) and degradation. The genes related to tapetum development, such as *EAT1* (Eternal Tapetum 1), *MIL2* (Microsporeless 2), and *DTM1* (Defective Tapetum and Meiocytese 1), were found to be involved in this process. When rice plants were exogenously sprayed with SA or paclobutrazol (PAC, a SA inhibitor) + H_2_O_2_ under heat stress, a significantly higher pollen viability was found compared to plants sprayed with H_2_O, PAC, or SA + dimethylthiourea (DMTU, an H_2_O_2_ and OH· scavenger). Additionally, a sharp increase in H_2_O_2_ was observed in the SA or PAC+ H_2_O_2_ treatment groups compared to other treatments.

**Conclusion:**

We suggest that H_2_O_2_ may play an important role in mediating SA to prevent pollen abortion caused by heat stress through inhibiting the tapetum PCD.

**Electronic supplementary material:**

The online version of this article (10.1186/s12870-018-1472-5) contains supplementary material, which is available to authorized users.

## Background

Rice (*Oryza sativa* L.) is one of the most important cultivated food crops worldwide, particularly in East and Southeast Asia, which belong to tropical and subtropical zones [1, 2]. In these areas, extremely high temperatures occur with increasing frequency during recent years [3]. It has been well documented that extremely high temperatures during the reproductive stage significantly decreases the grain yield by more than 50%, even causing complete loss of harvest in rice plants [4, 5]. Male sterility induced by heat stress is one of the main factors that cause the decrease in grain yield of rice. It has been reported that the young microspore stage, immediately after meiosis, shows sensitivity to high temperature [6]. Heat stress occurring at this stage causes the degeneration of microspores and the hypertrophy of tapetal cells, ultimately causes male sterility [7–9].

One of the essential steps of the sexual reproduction of plants is the production of viable pollen from anthers, which have four lobes with similar structure that are attached to a central core via connective and vascular tissues. Mature meiosis cells at the center of the anther lobe have four somatic layers: the epidermis, the endothecium, the middle layer, and the tapetum from the surface to the interior [10]. As the innermost sporophytic layer of the anther wall, the tapetum plays an essential role for the development from microspores to pollen grains. The tapetum is in direct contact with the developing gametophytes that provide enzymes for the release of microspores from tetrads as well as nutrients for pollen development [10, 11]. Mutants cannot produce viable pollen, which is partially due to defects in tapetum function [12, 13]. Such results have also been reported in plants that suffered from severe abiotic stress during the meiosis stage [8, 14, 15], since plants are particularly sensitive to abiotic stress during the meiosis and young microspore stage [14]. During this stage, even mild or short-term abiotic stress can cause a significant decrease in pollen fertility.

Sorghum and rice display male sterility if they are exposed to cold conditions during meiosis and microspore development, which mainly results in abnormal tapetum development and degradation, and ultimately leads to aberrant pollen [16]. In heat-susceptible rice plants, heat stress lasting 4 days or longer during the early phase of the anther development causes premature degradation of tapetum cells and programmed cell death (PCD), and leads to complete male sterility [17, 18]. Additionally, tapetum cells with a large vacuole were observed at the tetrad stage under heat conditions, while at the uninucleate stage, the membrane was blebbing and the cytoplasmic was in a state of degradation [19]. During this process, enlarged and irregularly shaped mitochondria as well as severely shrunken tapetal cells were also found in stressed plants, resulting in degeneration of the pollen exine layer and empty anther locules. Under heat stress, the genes related to male sterility (such as *YY1, YY2, TGMS*, and *tms5*) were down regulated in rice [19, 20]. Additionally, the *Arabidopsis YUCCA* gene exhibited down regulation under heat stress, especially in tapetum and pollen mother cells, which led to a significant decrease of male fertility [7]. Interestingly, changes of indole-3-acetic acid (IAA), gibberellins (GAs), abscisic acid (ABA), cytokinins (CTKs), free proline, and soluble protein contents were found in stressed anthers; however, except for the IAA, 6-benzylaminopurine (6-BA), ABA, and other phytohormones or regulators including salicylic acid (SA) have been rarely reported to reverse the pollen sterility caused by heat stress [7, 21, 22].

SA is known as a phenolic compound that naturally exists in plants at a very low concentration; however, it is a hormone-like substance that plays an important role for the regulation of plant growth and development [23, 24]. Increasing evidence suggests that SA not only functions in response to biotic stress [25, 26], but also plays an important role in abiotic stress, including cold [27, 28], drought [29], heavy metal [23, 30], and heat stress [3, 31, 32]. Khan et al. [31] reported that exogenous acetyl SA enhanced the thermo-tolerance in four-week-old tomato seedlings by improving root morphological features and root activity. Ca^2+^ homeostasis, antioxidant systems, H_2_O_2_, and hydrogen sulfide are involved in SA-induced heat tolerance of plants [33, 34]. Additionally, Clarke et al. [35] suggested that JA acted with SA inducing basal thermo-tolerance in *Arabidopsis thaliana*. These results indicate that SA can protect plants from heat damage during the vegetative stage, while only few reported studies focused on the reproductive stage. Our previous results indicated that SA could alleviate damage of the spikelet differentiation caused by heat stress during the floret differentiation stage of rice [3, 36]. Furthermore, SA has been suggested to be involved in plant flowering and pollen tip growth [37]. Thus, we suspect that SA can reduce pollen sterility when encountering high temperature stress at the PMC meiosis stage, which has not been documented before. To investigate the underlying mechanism, the pollen viability, tapetum ultrastructure, tapetum PCD, antioxidant enzymes, reactive oxygen species (ROS), and the expression of tapetum development genes were investigated.

## Methods

### Plant materials and growth conditions

The rice hybrid Changyou1, which was considered to be thermally susceptible, was purchased from the academy of agriculture sciences of Changshu city, Jiangsu province. Seeds were sown in buckets with 15 cm radius and 30 cm height at the China National Rice Research Institute, Hangzhou, China, during the period from May to September. The rice seeds were soaked for 48 h and then sprouted at 37 °C for 24 h. About 20–25 grains were sown per pot and then thinned to four plants when the fifth leaf was emerging. The pots were filled with 15 kg paddy soil with a sufficient amount of base fertilizer. Rice plants were cultivated in greenhouse with an automatic temperature control system to control the temperature until the PMC meiosis stage. The distance between the pulvinus of the 1st leaf and 2nd leaf from the top of the rice spikelets was about − 2 cm in the main tiller [38]. The following environmental conditions were applied: temperature of 30/24 °C, relative humidity of 70/80% (day/night), and natural sunlight.

### Effect of SA on plant tolerance to heat stress

At the PMC meiosis stage, rice plants were sprayed with different concentrations of SA: 0 (NON-SA treatment), 0.01, 0.1, 1.0, 10, and 50 mM (20 ml per pot) and then divided into two groups. One of them was subjected to high-temperature stress treatment of 40 °C for 10 d (heat-stress-duration from the PMC meiosis stage to 1 d before flowering, 40 °C from 09:00 to 15:00, with 30 °C at night), while the other group served as control (30 °C days and 24 °C at night) with the following environmental conditions: relative humidity of 70/80% (day/night), and natural sunlight. The spikelet containing anthers were harvested on day 4 and 10 after heat stress. Samples collected on day 4 were used for the observation of tapetum ultrastructure and PCD as well as the determination of relative expression of genes related to pollen development in anthers, while those samples collected on day 10 were used to determine the pollen viability, ROS, Caspase 3 activity, antioxidant enzyme activities, and MDA content. After heat stress treatment, rice plants were transferred back to the greenhouse under natural conditions until full maturity.

### The role of H_2_O_2_ for SA-induced heat tolerance

To investigate the role of H_2_O_2_ in the SA-induced heat tolerance of rice pollen, rice plants at the PMC meiosis stage were sprayed with SA, H_2_O_2_, paclobutrazol (PAC, a SA inhibitor, [39]), and dimethylthiourea (DMTU, an H_2_O_2_ and OH· scavenger, [40]) either alone or in combination, while distilled water was used as control before heat stress commenced. The following treatments were used: H_2_O, SA (10 mM), PAC (300 PPM), SA + DMTU (10 mM), and PAC + H_2_O_2_ (30 mM). The chemicals were sprayed onto rice plants every 2 days at 20 ml per pot. Spikelets containing anthers were harvested to determine the H_2_O_2_ and MDA content and antioxidant enzyme activities 7 h after chemicals were sprayed on day 9 after heat stress. Spikelets with anthers were also collected to determine pollen viability on day 10 after heat stress.

### Pollen viability measurement

Pollen viability was determined using the method of Gunawardena et al. [41]. Mature pollen grains collected from spikelets were stained with potassium iodide/iodine solution (KI/I_2_). Pollen grains were removed from anthers of the florets, placed into a drop of KI/I_2_ on a glass slide, and observed and photographed under a light microscope (DM4000B, Leica, Wetzlar, Germany). Ten replicates were investigated for the measurement of pollen viability.

### Quantification of reactive oxygen species

For both visualization and analysis of anther ROS, the oxidation-sensitive probe DCFH-DA was used, as previously described by Sanchez et al. [42]. Anthers were collected from the spikelets and were immediately incubated with 5 μM DCFH-DA. The fluorescence intensity was measured after 30 min of incubation with 5 μM DCFH-D via fluorescence microscopy (DM4000B, Leica, Wetzlar, Germany). The software Adobe Photoshop CS2 was used to determine the green brightness. Ten replicates were investigated for the observation of ROS.

### Quantification of hydrogen peroxide

According to the method of Brennan and Frenkel [43], frozen anthers (10 mg) were homogenized in 4 ml of 10 mM 3-amino-1,2,4-triazole, and then centrifuged for 25 min at 6000 *g* for 25 min at 4 °C. 2 ml of the supernatant were mixed with 1.5 ml 0.1% titanium tetrachloride, which was dissolved in 20% H_2_SO_4_. The reaction mixture was centrifuged, and the supernatant was monitored at 410 nm.

### Malonaldehyde measurement

According to the method of Dionisio-Sese and Tobita [44], frozen anthers (10 mg) were homogenized via 5 ml of trichloroacetic acid, and then 2 ml of the homogenate were added to 2 ml 0.6% (m/v) thiobarbituric acid. The reaction mixture was placed in a boiling water bath for 30 min and then placed in cold water for rapid cooling. Absorbances were measured at 450, 532, and 600 nm. MDA was calculated via the following formula: C (lM) = 6.45*(A532-A600)-0.56*A450.

### Measurement of antioxidant enzyme activities

Frozen anthers (10 mg) were ground into a fine powder in liquid nitrogen and then homogenized in 5 mL 50 mM sodium phosphate buffer (pH = 7.0). The homogenate was centrifuged at 1000 *g* for 15 min at 4 °C, and about 4 mL supernatant was stored in aliquots at − 20 °C until further analysis. The superoxide dismutase (SOD) activity assay was based on the inhibition of the photo reduction of nitroblue tetrazolium (NBT) as previously described by Giannopolitis and Ries [45]. The peroxidase (POD) activity assay was based on the conversion of guaiacol to tetraguaiacol, which was monitored at 470 nm, as previously described by Chance and Maehly [46]. The catalase (CAT) activity was measured according to the previously described method of Aebi [47] with some modifications. The ascorbate peroxidase (APX) activity was measured according to the method of Bonnecarrère et al. [48].

### Caspase 3 activity analysis

The caspase-3 activity was measured as previously described by Hu et al. [49] using the Caspase-3 activity kit (Beyotime Institute of Biotechnology, Haimen, PR China) following to the manufacturer’s protocol.

### TUNEL assay

For nuclear DNA fragmentation analysis of the tapetum, anthers were fixed and sectioned according to methods described by Vizcay-Barrena and Wilson [50]. The terminal deoxynucleotidyl transferase-mediated dUTP nick-end labeling (TUNEL) detection kit (Roche; Code: 11684817910) was used to determine the situ nick end labeling of nuclear DNA fragmentation according to the manufacturer’s protocol. Negative controls were conducted in the absence of the TUNEL enzyme. Positive controls were generated by incubating tissues with DNase I (Roche) for 10 min at 25 °C prior to labeling. The 2x SSC were used to stop the labeling reaction and the slides were rinsed with PBS (pH = 7.4). For fluorescence microscopy, the slides were counterstained with 2 μg·ml^− 1^ DAPI and remained at room temperature in the dark. Slides were rinsed thrice with PBS for 5 min each and then an epifluorescence microscope (NIKON DS-U3) was used to investigate TUNEL signals.

### Microscope analysis

For transmission electron microscopic (TEM) analysis, anthers were fixed with 2.5% glutaraldehyde in phosphate buffer (0.1 M, pH = 7.0) for more than 4 h, and then washed thrice in phosphate buffer (0.1 M, pH = 7.0) for 15 min. Finally, these specimens were post-fixed with 1% OsO_4_ in phosphate buffer (0.1 M, pH = 7.0) for 1–2 h and washed thrice in phosphate buffer (0.1 M, pH = 7.0) for 15 min. Prior to the dissection of individual anthers, the tissues were dehydrated in a graded ethanol series (30%, 50%, 70%, 80%, 90%, 95%, and 100%) for 20 min per step and slowly and progressively infiltrated with Spurr resin and mixed with acetone, achieving 100% Spurr’s resin overnight. Specimens were placed in an Eppendorf contained with Spurr resin and heated to 70 °C for more than 9 h. The specimens were sectioned in a LEICA EM UC7 ultramicrotome and sections were stained via uranyl acetate and alkaline lead citrate for 5 to 10 min, respectively, before they were observed in a Hitachi Model H-7650 TEM.

### Real-time PCR analysis

The three genes including *EAT1*, *MIL2*, and *DTM1* were chosen for analysis. Among these genes, *EAT1* is a basic helix-loop-helix transcription factor conserved in land plants, which positively regulates programmed cell death in tapetal cells in rice anthers [13]. *MIL2* is responsible for the differentiation of primary parietal cells into secondary parietal cells in rice anthers [51], while *DTM1* plays important roles in the ER membrane during early tapetum development [52]. The primers used for RT-PCR amplification are listed in Table 1. PCR reaction and detection were performed as previously described [53]. The 2^−ΔΔCT^ method was used to determine the relative gene transcript levels, using the mean value of triplicate experiments.

**Table 1:** Primer sequences for the three genes used in RT-PCR

Gene	MSU_Locus	Forward (5′-3′)	Reverse (5′-3′)
*EAT1*	LOC_Os04g51070	AAGGCCAACTCTCTGCTTCATG	AAACCGCCGAACCTTCTGATAC
*MIL2*	LOC_Os12g28750	GCCTCGTCATCCACCAGAAG	GACCTGTGTCGTCGTTGGAG
*DTM1*	LOC_Os07g43010	CGAAACGTCTAATGGGGATTGGG	GCTACTGAGATCAAGGGGAGGA
*UBQ5*	LOC_Os01g22490	GACTACAACATCCAGAAGGAGTC	TCATCTAATAACCAGTTCGATTTC

*EAT1*, Eternal Tapetum 1. *MIL2*, Microsporeless 2. *DTM1*, Defective Tapetum and Meiocytese 1. *UBQ5* was used as reference gene

### Seed-setting rate measurement

When mature, rice plants were harvested to determine the numbers of filled grain (FG) and abortive grain (AG) per panicle, and the seed-setting rate was calculated as FG/(FG + AG) × 100%.

### Statistical analyses

**Fig. 1:**
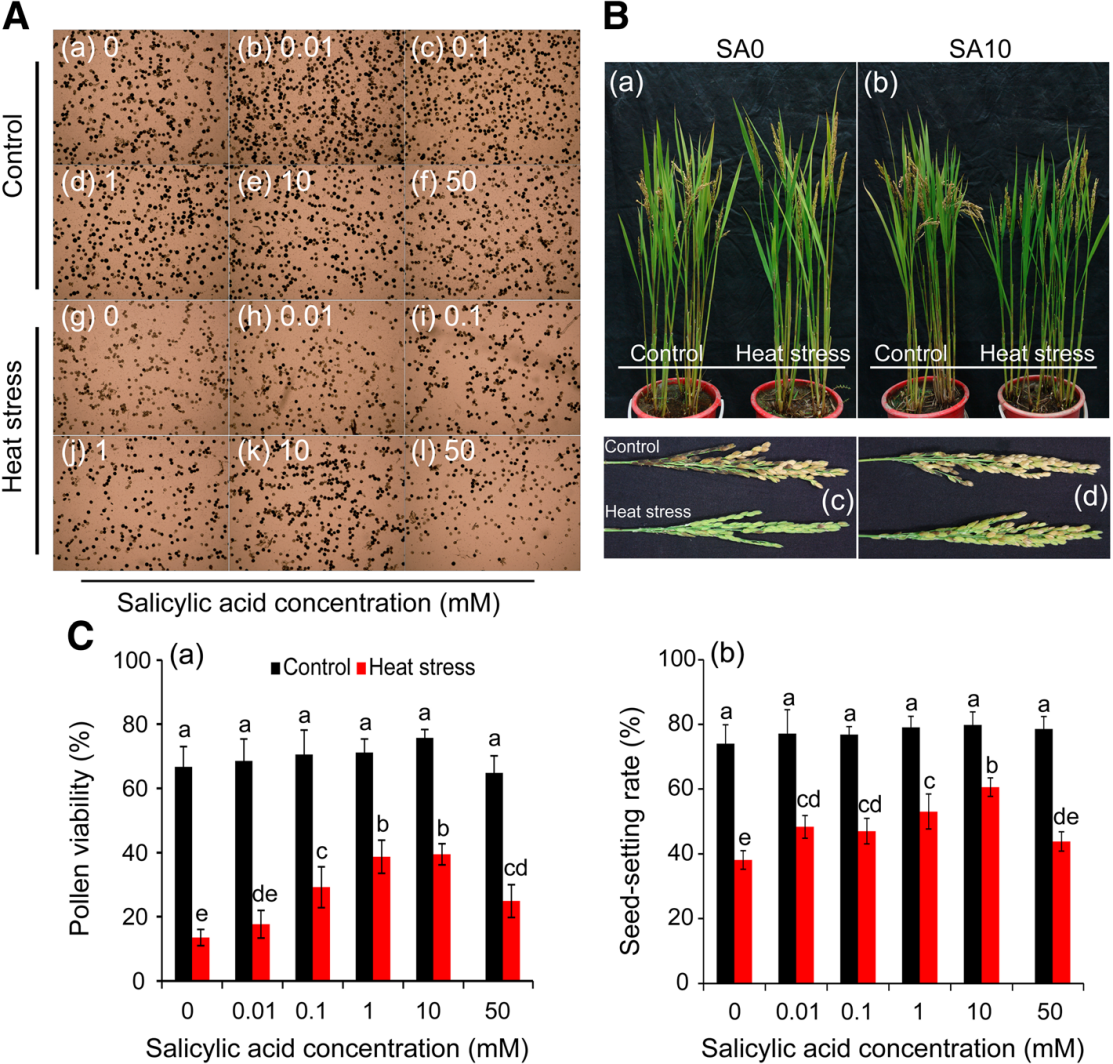
Effect of SA on the pollen viability and seed-setting rate of rice under heat stress at the pollen mother cell meiosis stage. **a**, the images of pollen grains in rice plants sprayed with salicylic acid under control and heat stress; a-f and g-i were the images of pollen grains of rice plants under control and heat stress, respectively. **b**, the images of rice plants with panicles sprayed with SA under control and heat stress. a and c, the images of rice plant sprayed with H_2_O (SA0); b and d, the images of rice plants and panicles sprayed with 10 mM SA (SA10). **c**, the data in the figure (a) and (b) were shown as the mean of ten and three replicates, respectively. Vertical bars denote standard deviations (*n* = 10 and 3 in (a) and (b), respectively). Different letters indicate significant differences between the SA treatments under control and heat stress (*P* < 0.05)

**Fig. 2:**
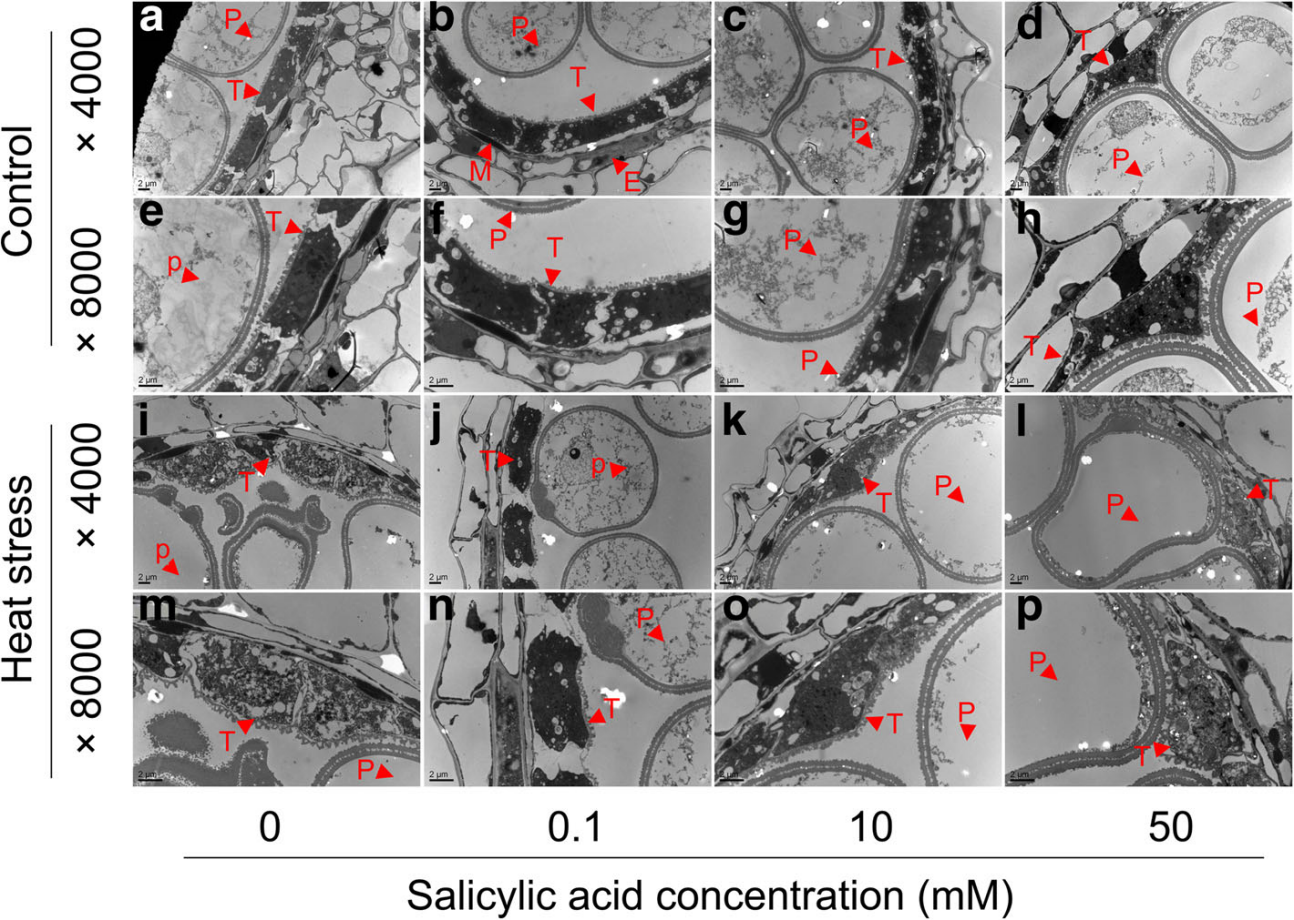
Transmission electron micrographs of cross-sections through anthers of rice plants at the vacuolization microspore stages sprayed with SA under control and heat stress. **a**-**h**, anthers under control pretreatment with different SA concentrations; **i**-**p**, anthers under heat stress pretreatment with different SA concentrations. Bar = 2μm. P, pollen grain; T, tapetum; M, middle layer; E, endothecium

**Fig. 3:**
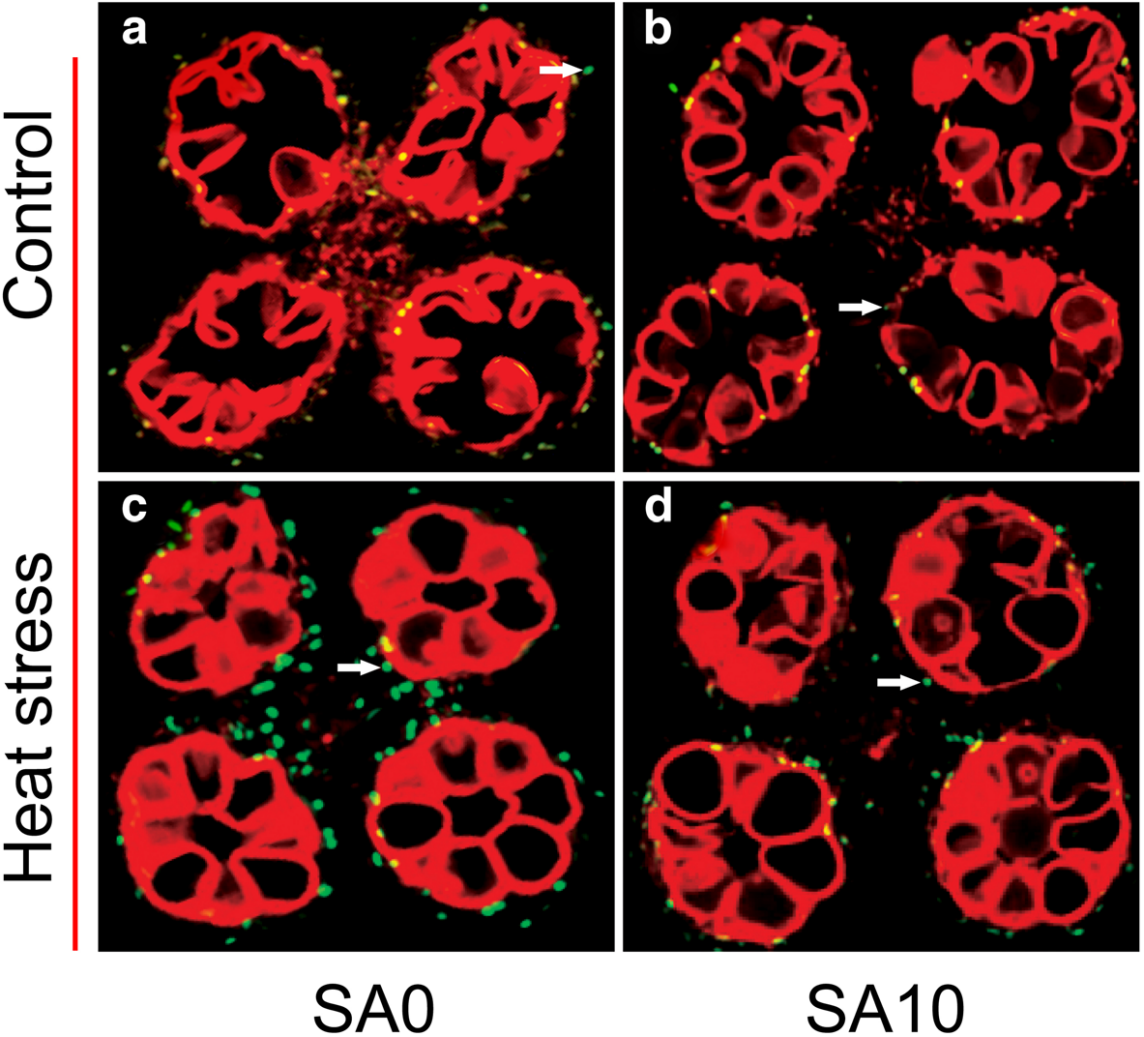
Detection of fragmented DNA via TUNEL assay in the tapetum of the anther under control and heat stress. TUNEL-positive signal was marked by a white arrow. **a** and **b**, control with 0 and 10 mM SA respectively; **c** and **d**, heat stress pretreatment with 0 and 10 mM SA respectively

**Fig. 4:**
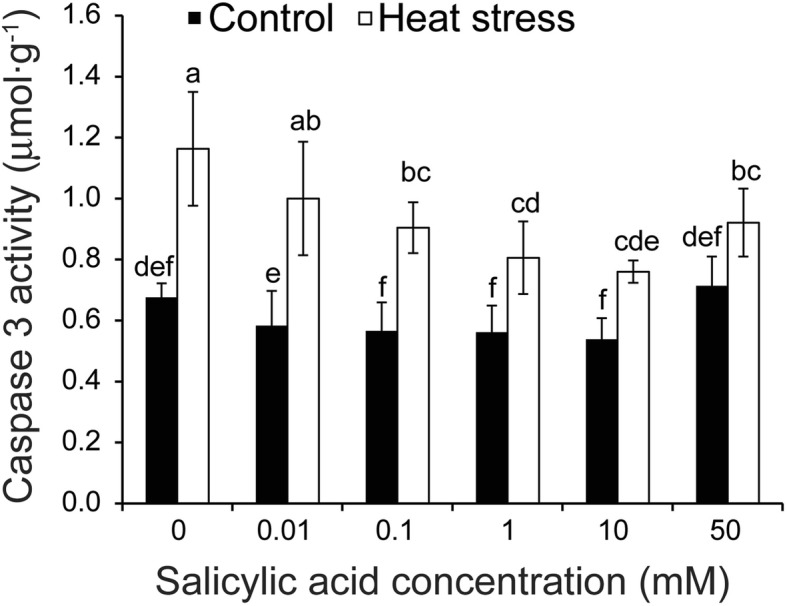
Effect of SA on the caspase 3 activity of anthers in rice plants in response to heat stress. Vertical bars denote standard deviations (*n* = 3). Different letters indicate significant differences between the SA treatments under control and heat stress (*P* < 0.05)

**Fig. 5:**
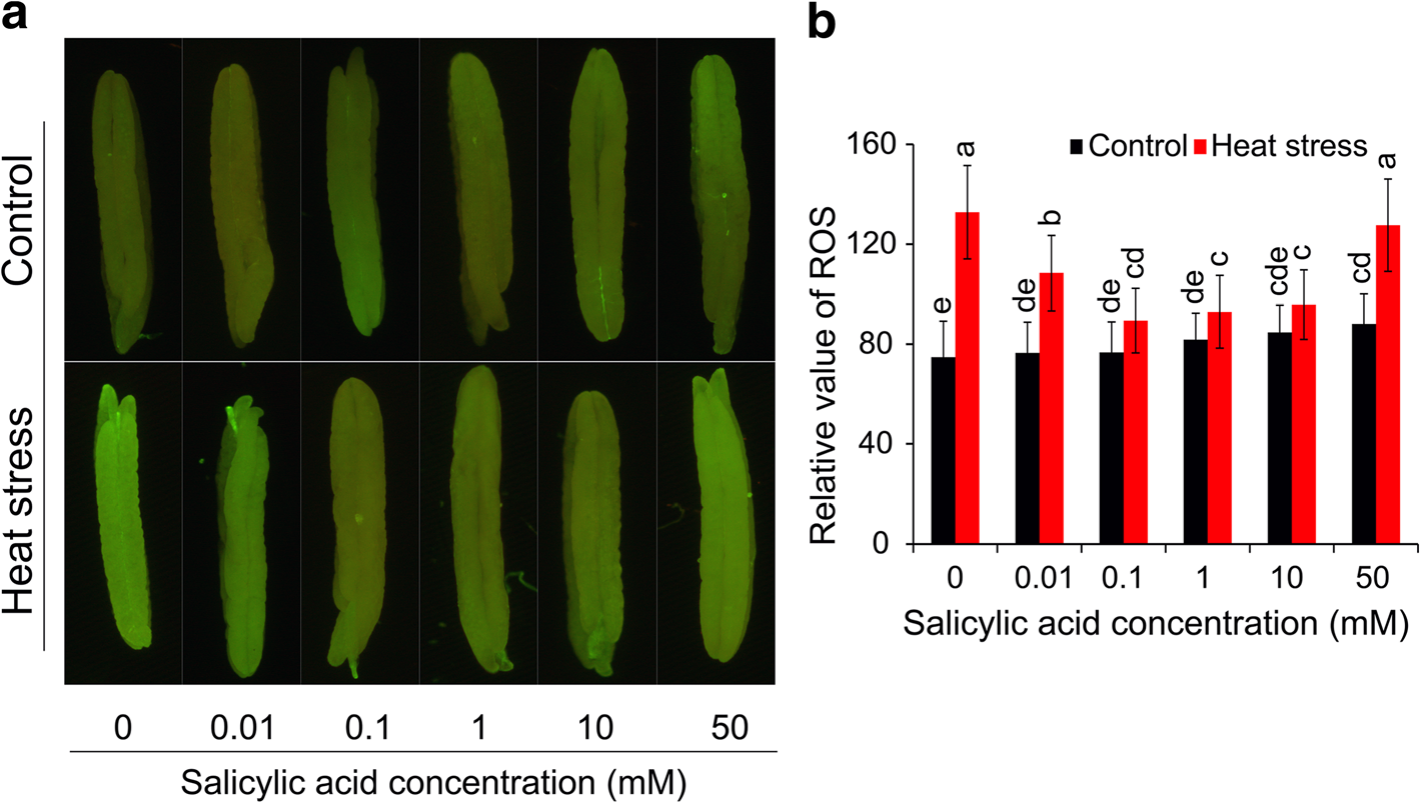
Effect of SA on the reactive oxygen species of anthers under control and heat stress. The anthers were incubated with 5 μM DCFH-DA, and were measured after 30 min by a fluorescence microscope. **a**, the fluorescence images of the anthers; **b**, these data were obtained from the fluorescence images (*n* = 10). Vertical bars denote standard deviations (*n* = 10). Different letters indicate significant differences between SA treatments under control and heat stress (*P* < 0.05)

**Fig. 6:**
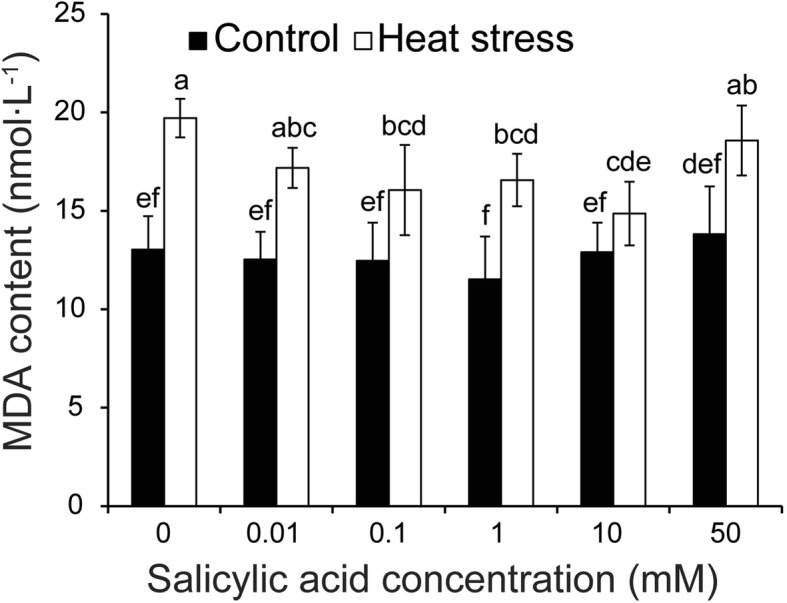
Effect of SA on the MDA concentration of anthers in response to heat stress. Vertical bars denote standard deviations (*n* = 3). Different letters indicate significant differences between the SA treatments under control and heat stress (*P* < 0.05)

**Fig. 7:**
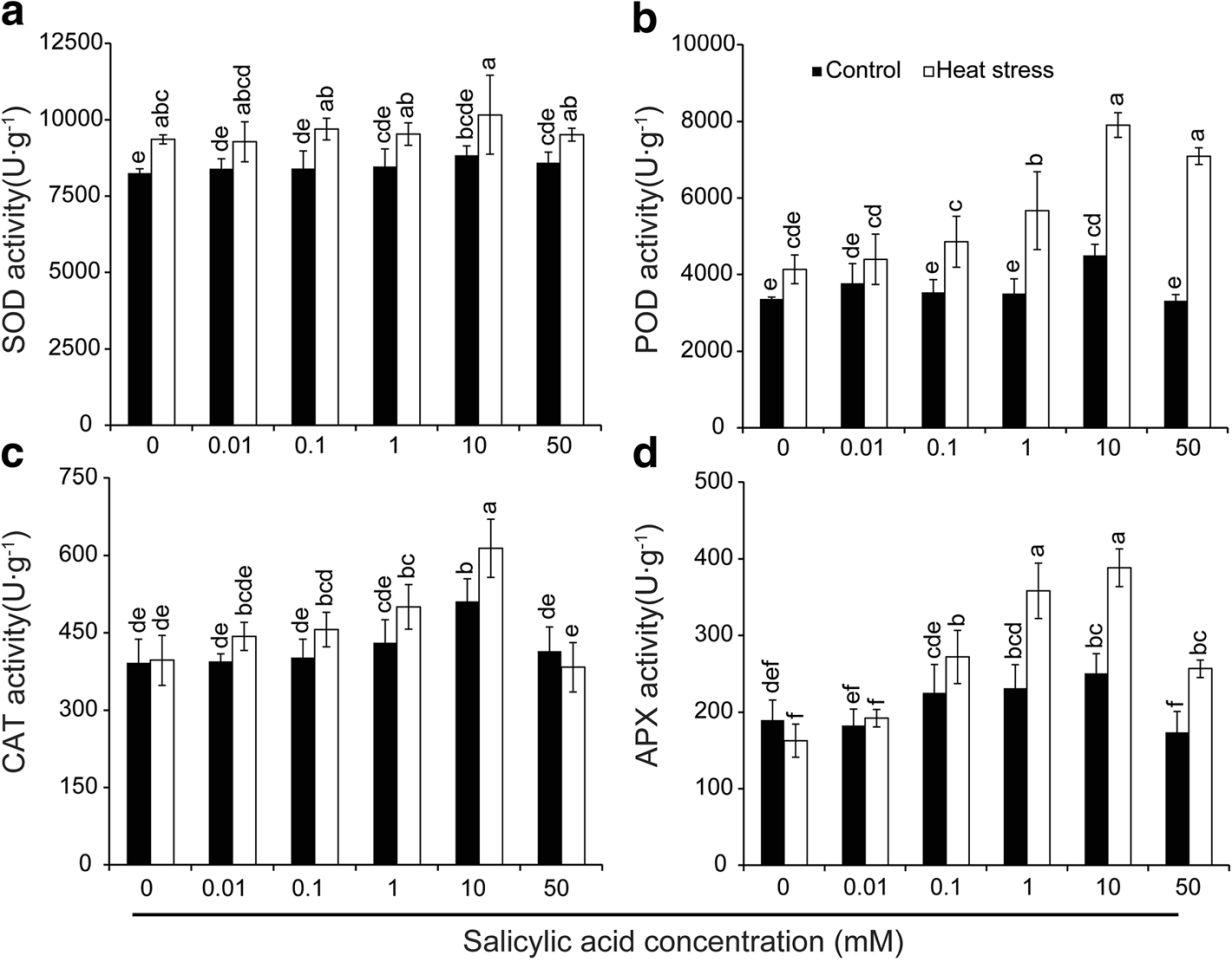
Effect of SA on activities of the antioxidant enzyme including SOD (**a**), POD (**b**), CAT(**c**) and APX (**d**) in anthers of rice under control and heat stress. Vertical bars denote standard deviations (*n* = 3). Different letters indicate significant differences between the SA treatments under control and heat stress (*P* < 0.05)

**Fig. 8:**
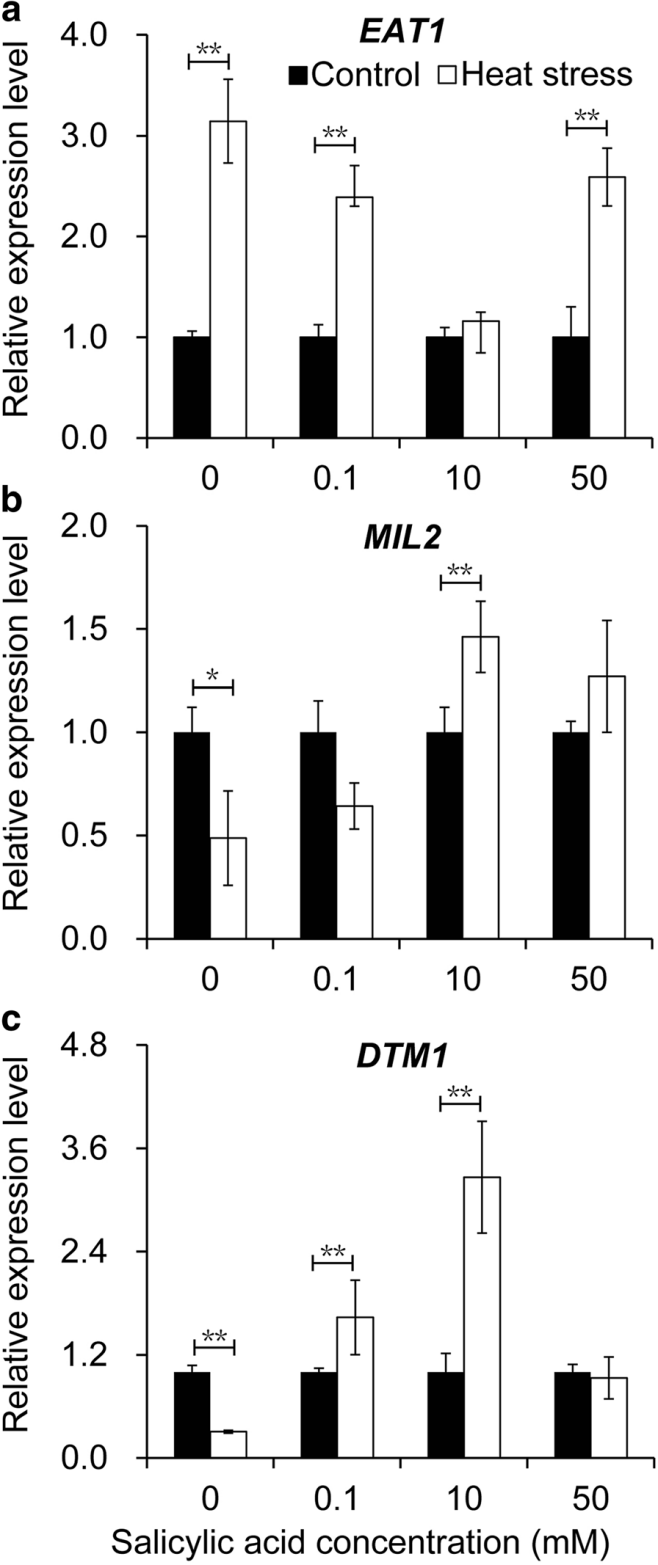
Effects of SA on the expression levels of tapetum development genes in rice anther in response to heat stress. **a**, *EAT1* gene (Eternal Tapetum 1); **b**, *MIL2* gene (Microsporeless 2); **c**, *DTM1* gene (Defective Tapetum and Meiocytese 1). Vertical bars denote the standard deviation (*n* = 3). A t-test is conducted to compare difference between control and heat stress. * denotes *P* < 0.05, ** denotes *P* < 0.01

**Fig. 9:**
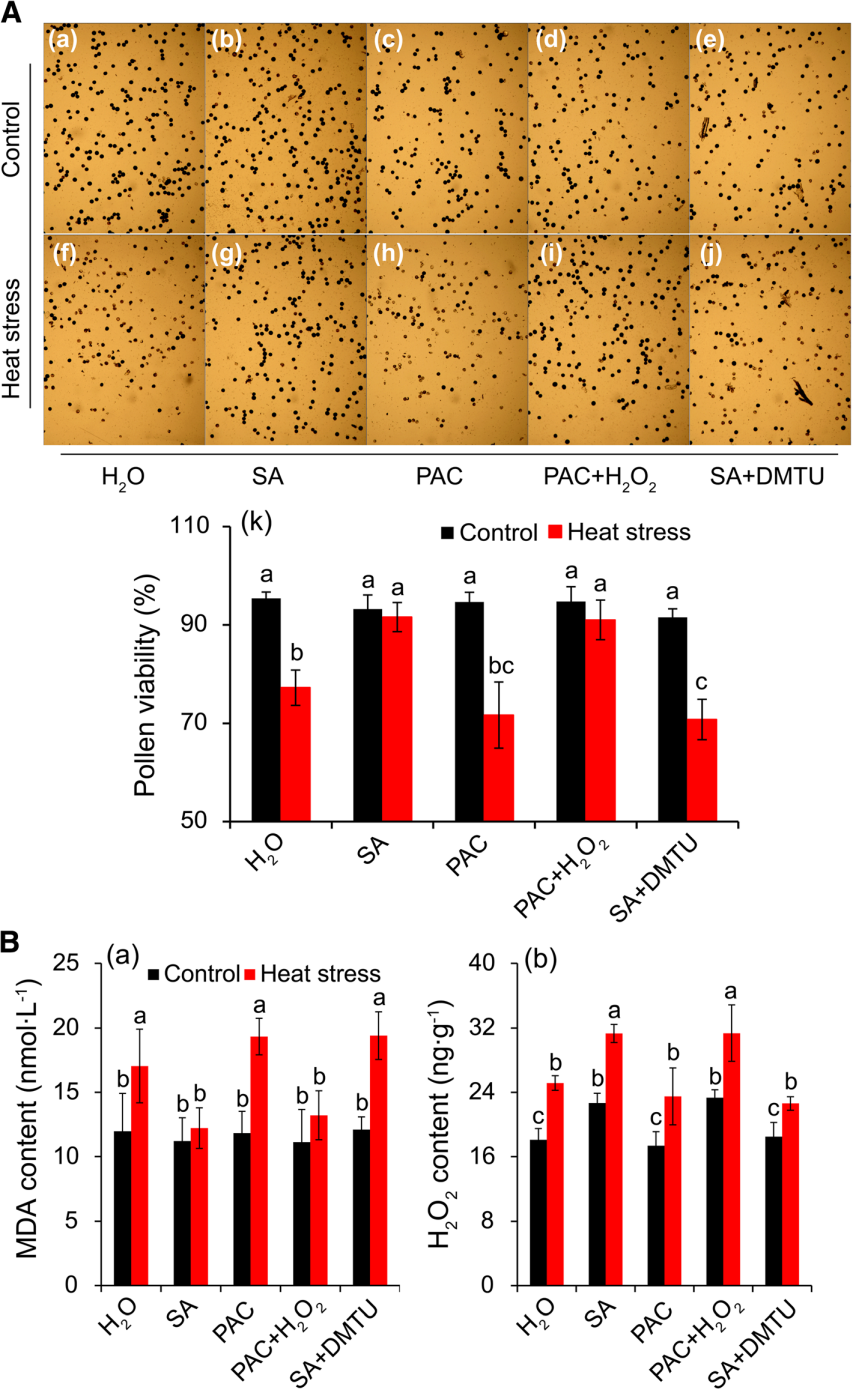
The changes in pollen viability, MDA and H_2_O_2_ of rice plants sprayed with H_2_O, SA, DMTU, H_2_O_2_, and PAC along or together under control and heat stress. A-(a-j), the photos of pollen grains was stained with 1% with KI/I_2_ by a microscope (Leica, DM4000). A-(a-e), pollen grains under control condition. A-(f-j), pollen grains under heat stress condition. B-a and B-b, MDA and H_2_O_2_ at anther respectively. The data in the Fig. (A-k) were shown as the mean of ten replicates and Fig. (B-a and b) were shown for three replicates. DMTU, dimethylthiourea, an H_2_O_2_ and OH· scavenger. PAC, paclobutrazol, a SA inhibitor. Different letters indicate significant differences between treatments under control and heat stress (*P* < 0.05)

Data were processed with SPSS (version 11.5) and Excel 2010 software. The mean values and standard deviations shown in the figures represent the pooled data of three replicates unless otherwise stated. Two-way ANOVA for two factors (temperature and treatment) was conducted to compare the difference with a least significant difference test (LSD) at *P* < 0.05 for the data in Figs. 1, 2, 3, 4, 5, 6, 7 and 9. A t-test was conducted for data in Fig. 8 to compare the difference between control and heat stress. * denotes *P* < 0.05, ** denotes *P* < 0.01.

## Results

### Effect of SA on pollen viability and seed-setting rate under heat stress

Under control conditions, no significant difference was found in pollen viability among SA treatments (Fig. 1A, a-f, and C-a). However, an average decrease in pollen viability of 60.8% was observed in plants under heat stress compared to the control (Fig. 1B, g-I, and C-a). Rice plants sprayed with SA of 0.1 and 1.0 mM attained significantly higher pollen viability than plants of the NON-SA treatment under heat stress. Decreases of 58.6%, 45.6%, and 47.9% were recorded for SA treatments of 0.1, 1.0, and 10 mM, respectively, while a decrease of 78.8% was observed in the NON-SA treatment group compared to their respective controls (Fig. 1b). Additionally, the pollen viabilities in the SA treatments of 0.01 and 50 mM were slightly higher than that of NON-SA treatment under heat stress.

The seed-setting rates ranged between 74 and 80% under control conditions and no significant differences were found among the SA treatments (Fig. 1B and C-b). Under heat stress, the seed-setting rates deceased significantly, particularly for the NON-SA treatment, where values were clearly lower than those of plants sprayed with SA except for the treatment of 50 mM. Compared to the control, the seed-setting rate of NON-SA treatment deceased by 48.5% under heat stress, while decreases of only 32.9 and 24.1% were found in rice plants sprayed with 1.0 and 10 mM SA, respectively. Interestingly, a decrease of about 44.2% in seed-setting rate caused by heat stress was observed in the 50 mM SA treatment, which was slightly lower than that observed in the NON-SA treatment.

### Effect of SA on ultrastructural features of tapetum in anthers under heat stress

Pollen abortion caused by heat stress might be related to abnormal development of the tapetum in anthers. TEM was performed to obtain a more detailed understanding of these abnormalities. Anthers were collected at the vacuole microspore stage when the middle layer was not visible and the tapetum started to degenerate. Ultrastructural studies demonstrated that complete tapetum tissues were still visible in the anthers in control conditions, irrespective of SA concentrations (Fig. 2a-h). Degenerated tapetum tissues were found in the anthers under heat stress (Fig. 2i-p); however, the degree of damage depended on SA concentrations. The tapetum tissues in anthers of rice plants sprayed with 0 and 50 mM were severely damaged under heat stress. However, rice plants sprayed with 0.1 and 10 mM could effectively alleviate this damage, in particular the latter, in which a relatively complete ultrastructure was still visible (Fig. 2k and o).

### Effect of SA on TUNEL assay of tapetum cells apoptosis and Caspase 3 activity under heat stress

In plants, PCD is the main factor that leads to tapetal cell degeneration, manifesting as cell condensation, nuclear degeneration, membrane breakdown, and the cleavage of nuclear DNA [13]. To investigate the abnormal degeneration in the heat-stressed tapetum, a TUNEL assay was performed. In general, the TUNEL-positive signals in the tapetum of non-stressed anthers were weaker than those subjected to heat stress (Fig. 3). Under control conditions, no significant difference was detected between the NON-SA and SA10 treatments (Fig. 3a-b); however, under heat stress, positive signals in rice plants sprayed with 10 mM were significantly weaker than plants sprayed with H_2_O (Fig. 3c-d).

Since tapetum cell apoptosis could be induced by Caspase 3 activity under heat stress, we used the Caspase 3 Assay Kit to determine its activity in anthers. Without exception, little difference was found in the Caspase 3 activity among SA treatments under control conditions, although a slight increase was observed in rice plants sprayed with SA of 50 mM (Fig. 4). Caspase 3 activity was noticeably increased by 52.7% in anthers of stressed plants compared to non-stressed plants. Under heat stress, the highest Caspase 3 activity was observed in the NON-SA treatment, which was noticeably higher than those in the SA treatments except for the treatment of 0.01 mM SA. The lowest activity was found in plants sprayed with 10 mM SA, followed by the 1.0 and 0.1 mM treatments. Compared to their respective controls, an increase of 72.1% was observed in the NON-SA treatment under heat stress, while increases of 59.8%, 43.5%, and 41.3% were observed in the 0.1, 1.0, and 10 mM treatments, respectively. Interestingly, there was only a 29.1% increase in the 50 mM treatment under heat stress compared to control.

### Effect of SA on ROS, MDA, and antioxidant enzyme activities under heat stress

Anthers were incubated with 5 μM DCFH-DA to determine ROS via fluorescence. Few differences were found in the fluorescence intensity between the NON-SA and SA treatments except for the 50 mM treatment, which was significantly higher than that of NON-SA treatment (Fig. 5). Under heat stress, an average increase of 27.9% in ROS was found compared to that of non-stressed anthers. Under this condition, the highest ROS was observed in the anthers of the NON-SA treatment group, followed by 50 mM and 0.01 mM treatments, which were significantly higher by 77.5%, 44.8%, and 41.8% than their respective controls. However, only 16.5%, 13.6%, and 13.2% increases were found in the 0.1, 1, and 10 mM treatments under heat stress compared to their respective controls.

With regard to the MDA content in anthers, no significant difference was found among all treatments under control conditions, although the values were slightly higher in SA treatments of 10 and 50 mM (Fig. 6). Under heat stress, the MDA content of anthers was significantly increased compared to their respective controls, except for the 10 mM SA treatment. Under this condition, the highest MDA content was observed in the NON-SA treatment, which was significantly higher than those of SA treatments at 0.1–10 mM. However, the difference between the NON-SA treatment and SA treatments of 0.01 and 50 mM was not significant under heat stress.

The activities of antioxidant enzymes, such as SOD, POD, CAT, and APX, were also determined in anthers. Irrespective of control conditions or heat stress, little difference in SOD activity was observed among all treatments, although slightly higher activities were found in stressed anthers compared to non-stressed anthers (Fig. 7a). Under the control condition, no significant differences were found in POD activity among all treatments except for the treatment of 10 mM SA, which was significantly higher than those of the other treatments (Fig. 7b). POD activity was increased by heat stress, in particular for the SA treatments of 10 and 50 mM. With regard to CAT activity, a significant increase was found in anthers sprayed with 10 mM SA under both control and heat stress compared to NON-SA treatment (Fig. 7c). However, except for the 10 mM SA treatment, the difference among other treatments was not significant. A similar changing pattern was observed in the APX activities under control or heat stress, in which the highest activity was observed in the 10 mM SA treatment, followed by 1.0 and 0.1 mM SA treatments (Fig. 7d). Under heat stress, the APX activities of rice plants sprayed with SA at 10, 1.0, and 0.1 mM were significantly higher than that of the NON-SA treatment group. However, under control conditions, no noticeable difference in activity was observed between the NON-SA and SA treatments except for the 10 mM treatment. Interestingly, a significant increase in APX activity was induced by heat stress in plants sprayed with SA of 1.0–50 mM, while a slight decrease was found in NON-SA treatment compared to their respective controls.

### Effect of SA on expression levels of genes related to the tapetum development of rice under heat stress

The expressions of genes related to the tapetum development (*EAT1, MIL2*, and *DTM1*) were determined in anthers. A remarkable increase in the expression level of *EAT1* was found in rice anthers under heat stress compared to those under control except for the 10 mM SA treatment (Fig. 8a). Under heat conditions, the highest increase was found in the NON-SA treatment, followed by the treatments of 50 mM and 0.1 mM SA compared to their respective controls. Regarding *MIL2*, its expression in the NON-SA treatment was significantly decreased in response to heat stress compared to the control (Fig. 8b). However, this decrease was reversed by SA under heat stress, particularly for the 10 mM SA treatment, which was obviously higher than that of the control (Fig. 8b). Similarly, the expression of *DTM1* was also significantly decreased by heat stress when rice plants were treated with H_2_O under heat stress compared to the control (Fig. 8c). Interestingly, significant increases were found in plants treated with 1 and 10 mM SA when exposed to heat stress. However, no obvious difference was found between control and heat stress in response to the treatment at 50 mM. Furthermore, three additional genes were also detected in rice anthers: *DTC1*, *TDR* and *Udt1*. No significant differences in their expression levels were found among treatments (Additional file 1: Figure S1).

### Changes in pollen viability, H_2_O_2_, and MDA of anthers in rice plants sprayed with H_2_O, SA, PAC, H_2_O_2_, and DMTU under heat stress

According to previous results, H_2_O_2_ might be involved in mediating SA to alleviate damage of plants caused by heat stress. Thus, rice plants were sprayed with H_2_O, SA, PAC, PAC + H_2_O_2_, and SA + DMTU under both control and heat stress conditions during the PMC meiosis stage to investigate whether H_2_O_2_ was the mediator in this process. Under control conditions, pollen viabilities in all treatments ranged between 91 and 96% (Fig. 9A, a-e, and k). Accordingly, the difference among these treatments was not significant. Pollen viability was significantly decreased by heat stress, in which a decline of about 19.1% was recorded in anthers sprayed with H_2_O compared to the control, while declines of only 1.8% and 3.9% were found in anthers sprayed with SA and PAC + H_2_O_2_, respectively (Fig. 9A, f-j, and k). Without exception, the treatments of PAC and SA + DMTU, which were used to scavenge SA and H_2_O_2_, respectively, attained lower pollen viabilities compared to H_2_O treatment under heat stress, in particular the SA + DMTU treatment. Decreases of about 24.3% and 22.7% were observed in stressed-anthers sprayed with PAC and SA + DMTU compared to their respective controls.

Under control conditions, no significant difference was observed in MDA content among all treatments (Fig. 9B, a). The MDA content in anthers was significantly increased in response to heat stress when rice plants were sprayed with H_2_O, PAC, and SA + DMTU compared to their respective controls; however, no significant difference was found between control and heat-stressed anthers when rice plants had been treated with SA and PAC + H_2_O_2_. Accordingly, the highest MDA contents were found in the treatment of SA + DMTU, followed by treatments of PAC and H_2_O under heat stress, which were significantly higher than those of anthers sprayed with SA and PAC + H_2_O_2_.

With regard to H_2_O_2_, the highest contents in anthers were observed in plants sprayed with SA and PAC + H_2_O_2_ under control conditions, which was obviously higher than those of plants sprayed with H_2_O, PAC and SA + DMTU (Fig. 9B, b). Remarkable increases in H_2_O_2_ content were found in all treatments under heat stress compared to their respective controls. Similarly, the highest contents were found in the treatments of SA and PAC + H_2_O_2_ under heat stress, which were significantly higher than the levels of plants treated with H_2_O, PAC and SA + DMTU.

## Discussion

SA has been widely reported to confer resistance to plants under both biotic and abiotic stresses. SA concentrations are low, mainly ranging between 0.01 and 0.5 mM [54, 55]. However, such effects were not found at higher SA concentrations. In contrast, higher SA concentrations significantly inhibit the plant development irrespective of experimental conditions [25, 56]. Interestingly, the presented results indicate no obvious difference in the pollen viability and seed-setting rate among the SA treatments under control conditions (Fig. 1). Furthermore, under heat stress, rice plants sprayed with SA at 0.1–10 mM attained a significantly higher seed-setting rate and pollen viability compared to those of the NON-SA treatment (Fig. 1). The most effective SA concentration was found to be 10 mM under heat stress and no inhibition was found on the rice plants when they were sprayed with 50 mM SA. Although similar results have been reported in our previous study [3], the present SA concentrations were much higher than those reported in previous studies [57]. Such a phenomenon might be caused by the plant species, developmental stage, and environmental conditions. For plants, a low level of SA (0.1–0.5 mM SA) is optimal to elicit the highest level of stress tolerance [58]. Pretreatment with SA at these concentrations (0.1–0.5 mM) causes low levels of ROS accumulation in rice plants containing higher endogenous SA [59]. Additionally, our previous results indicated that 0.1 mM SA sprayed on rice plants at the floret differentiation stage noticeably alleviated the inhibition on the spikelet numbers caused by heat stress; however, no significant differences were found among SA treatments between 0.1–10 mM [3]. This phenomenon was also described by Kumar et al. [60], who suggested spraying of SA at 100 mM prior to heat stress as a most effective treatment in wheat cultivars that face heat stress. However, when we added SA to the nutrient solution, the rice plants were severely inhibited at higher concentrations compared to control conditions. Additionally, SA was reported to negatively affect the response to salt stress in pea plants [61]. Interestingly, such an inhibition was not found in rice plants grown in paddy fields, and even those sprayed with SA of 100 mM at the floret differentiation stage.

Pollen abortion of plants caused by abiotic stress is always a result of their abnormal tapetum development [26], which directly contacts the developing male gametophyte and plays a critical role in the development and maturation of microspores [10]. However, this tissue is susceptible to abiotic stress, especially during the PMC meiosis stage [62]. Drought stress at this stage significantly decreases pollen viability, which is possibly caused by the repression of the anther cell wall invertase gene (*IVR1*), leading to sucrose accumulation [63] and abnormal starch levels in a further connective tissue, ultimately resulting in pollen abortion [64]. Under cold stress, ABA increases and negatively regulates the expression of tapetum cell wall bound invertase and monosaccharide transport genes, resulting in a distorted carbohydrate pool in the anther and thus, in pollen sterility [65]. Without exception, SA reversed the heat stress caused pollen abortion of rice plants mainly through protecting tapetum tissues from heat damage. Under control conditions, the tapetum tissues in anthers of rice plants were complete irrespective of SA concentrations, suggesting that these tissues suffered little damage (Fig. 2a-h). However, the tapetum tissues were severely damaged by heat stress and displayed cell shrinkage, nuclear condensation, and fragmentation, especially for NON-SA treatment (Fig. 2i and m). Although increasing evidence confirms that SA enhances heat resistance in plants [32, 33], this protective function of SA on tapetum tissues has not been reported before. Since tapetum development involves an array of events, many factors including the genes related to tapetum development are involved in the dysfunction of tapetum caused by abiotic stress [66, 67]. Indeed, SA-mediated prevention of tapetum degradation caused by heat stress might be related to the genes *MIL2* and *DTM1*. *MIL2* regulates the differentiation of primary parietal cells into secondary parietal cells in rice anthers [51], while *DTM1* controls the early stage of tapetum development of rice [52]. In the present study, heat stress significantly inhibited the expression of *MIL2* and *DTM1* in anthers treated with H_2_O, while this inhibition was reversed by SA treatment. Significant decreases or clear increases were found in plants treated with specific concentration of SA under heat stress compared to their respective controls (Fig. 8b and c). However, how SA affects the expression of *MIL2* and *DTM1* under heat stress remains unclear.

PCD is a cell controlled and organized destruction process where cells are selectively eliminated in a highly coordinated and multi-step fashion through the involvement of specific proteases and nucleases [68]. PCD plays an important role during development and also during the stress response in plants [13, 69, 70]. However, PCD can also be a consequence of severe abiotic and biotic stresses. Increasing evidence indicates that apoptotic-like PCD can be induced by both biotic and abiotic stresses [71]. In this study, PCD was found in the tapetum under heat stress (Fig. 3), accompanied by tapetum tissue degeneration (Fig. 2), suggesting that PCD was a consequence of heat damage. SA could alleviate such damage, since the PCD in the tapetum of anthers that were sprayed with SA of 10 mM was noticeably lower than that of NON-SA treatment under heat stress (Fig. 3). This finding agrees with the result of Zhang and Chen [72], who reported that SA prevented Cd-induced photosynthetic damage and cell death, and the inhibition of ROS overproduction was the main factor. Interestingly, similar results were also found in the present research, in which the anther ROS of plants sprayed with H_2_O were significantly higher than those of plants sprayed with SA at 0.1–10 mM under heat stress (Fig. 5). Accordingly, their POD and APX activities were noticeably higher than that of NON-SA treatment (Fig. 7b and d). This suggests that under heat stress SA can enhance the antioxidant ability of rice plants to maintain the redox state by scavenging excess ROS, which is mainly contributing to PCD [68]. In general, lower doses of ROS are utilized as signals, mediating at least part of the responses to stresses; however, they pose a significant threat that may eventually lead to PCD at higher concentrations [73].

Under natural or abiotic stress, SA induces PCD through increasing H_2_O_2_, accompanied by a reduction of CAT and POD activities [61, 74]. This suggests that an antagonistic mechanism exists between SA and antioxidant enzymes such CAT, POD, and APX [61]. However, no significant difference was found in the activities of antioxidant enzymes among SA treatments under control conditions, while heat stress induced POD and APX activities of rice plants sprayed with SA at 1.0–10 mM were significantly higher than that of the NON-SA treatment (Fig. 7). H_2_O_2_ may be involved in this process that mediates SA to enhance heat tolerance in rice plants. This assumption was confirmed by the results obtained in this study, as rice plants sprayed with SA or PAC + H_2_O_2_ attained noticeably higher pollen viability than those plants sprayed with H_2_O, PAC, or SA + DMTU under heat stress (Fig. 9A). Accordingly, sprayed with SA or PAC + H_2_O_2_ showed a higher H_2_O_2_ content as well as lower MDA content compared to other treatments (Fig. 9B, a and b). This finding is consistent with the results of Dat et al. [33], who reported that thermos-protection can be obtained by spraying SA through an early increase in H_2_O_2_. Then, both H_2_O_2_ and catalase activity significantly decreased below control levels between 2 and 3 h after SA treatment, which occurred during the period of maximum thermos-protection. For this case, the low ROS accumulation induced by SA does not lead to cell death. In contrast, the non-toxic elevation of ROS content served as a signal for the activation of defensive responses in the cell, including enhancing its antioxidant abilities.

Currently, a novel family of cysteine proteases called metacaspases appeares to act as a trigger in ageing and oxidative stress-activated PCD. Caspase-3 activation is a landmark event in apoptosis [75]. Caspase-3 activation and caspase-3-like activity have been widely used as a definitive way of detecting PCD in plants [76]. The relationship between caspase-3 activity and SA remains unclear under control conditions since there was no noticeable difference in caspase-3 activity among SA treatments (Fig. 4). However, caspase-3 activity was inhibited by 0.1–10 mM SA under heat stress (Fig. 4). Similar changing patterns were also found in ROS, suggesting that it might mediate SA to inhibit the caspase-3-like activity of anthers of rice plants under heat stress (Fig. 3), since ROS requires caspase-like activation to cause cell death [77]. However, several genes involved in PCD during the tapetum degeneration in meiosis or post meiosis have been identified [13, 69, 78]. It is therefore possible that these genes can be directly induced by SA to regulate tapetum degeneration under heat stress, (e.g. *EAT1*). A significant increase induced by heat stress was found for the expression level of *EAT1*, while SA inhibited its expression under heat stress, in particular for the treatment of 10 mM (Fig. 8a). No significant difference in the expression level of *EAT1* was found between the control and heat stress in anthers sprayed with 10 mM SA (Fig. 8a). This pathway might be independent of ROS, possibly because no reports have indicated that ROS were required for the induction of those genes. However, the mechanism underlying SA inhibiting *EAT1* expression in anther under heat stress remains unclear.

## Conclusion

**Fig. 10:**
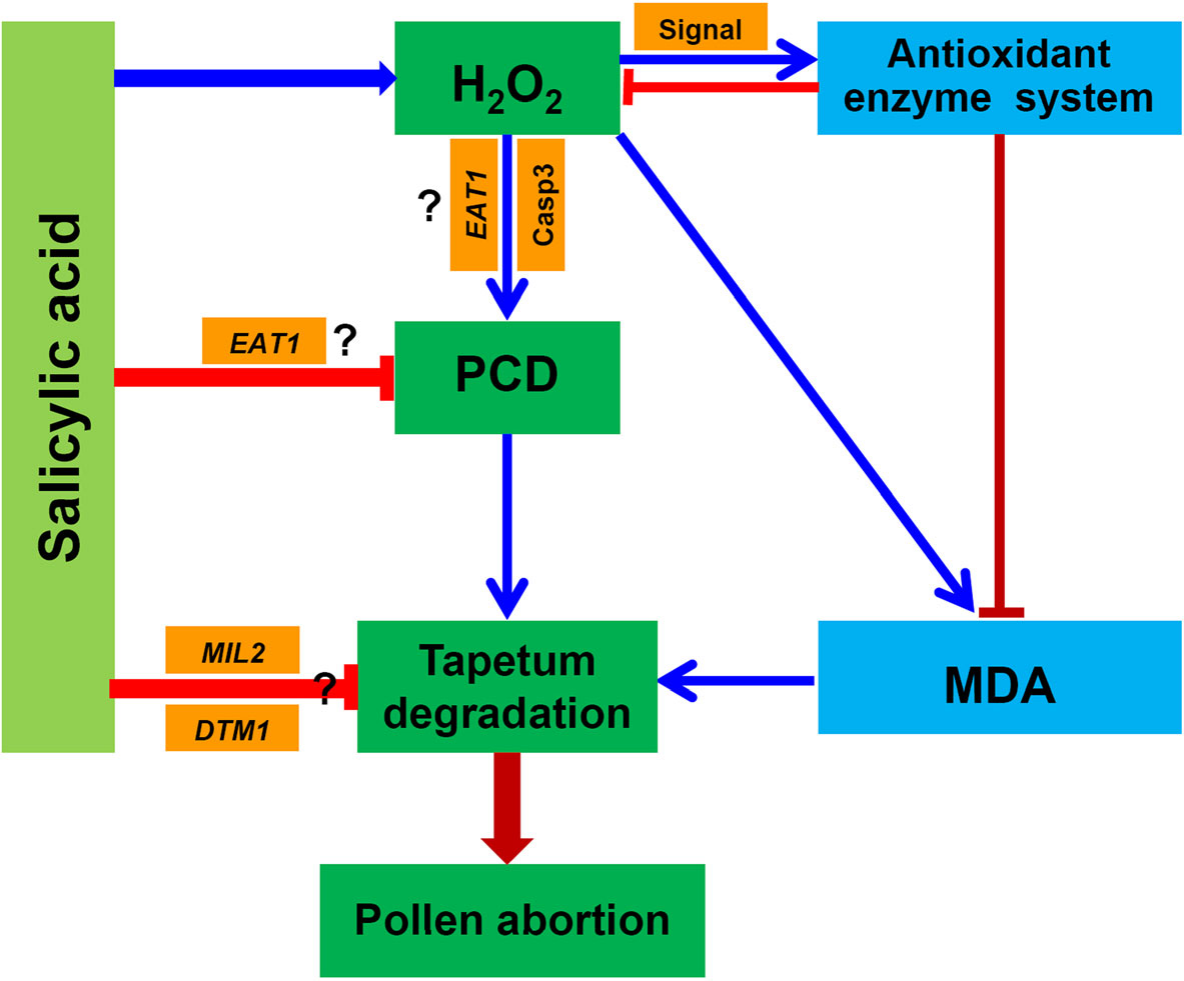
Descriptive model of the salicylic acid functions toward preventing pollen abortion of rice in response to heat stress. Under heat stress, H_2_O_2_ is significantly increased in anthers induced by SA under heat stress, which in turn enhances the antioxidant capacity to scavenge excessive ROS. This can inhibit PCD in anthers, and thus prevent tapetum degradation caused by heat stress. Genes such as *EAT1*, *MIL2*, and *DTM1* are involved in the process of SA-preventing tapetum degradation caused by heat stress, which may be independent of H_2_O_2_. H_2_O_2_, hydrogen peroxide; MDA, Malondialdehyde; Casp3, caspase 3 activity; *MIL2*, Microsporeless 2; *EAT1,* Eternal Tapetum; *DTM1*, Defective Tapetum and Meiocytese. The arrow mark “→” indicates induction, while “⊣” indicates inhibition

SA conferred heat resistance in rice plants at the PMC meiosis stage, where higher pollen viability and seed-setting rates were found in rice plants treated with SA under heat stress. This effect was mainly caused by the SA-mediated reduction of excessive ROS in anthers, which prevented tapetum degradation caused by heat stress by inhibiting the caspase-3 activation, and thus, PCD in anthers (Fig. 10). Additionally, genes related to tapetum development of rice such as *EAT1*, *MIL2*, and *DMT1* were found to be involved in SA-mediated prevention of heat stress caused tapetum degradation, although the underlying mechanism remains unclear (Fig. 10). This pathway might be dependent of H_2_O_2_, suggesting that SA could enhance the heat tolerance of rice plants through other pathways (Fig. 10). However, higher pollen viability and H_2_O_2_ levels as well as lower MDA levels were found in rice plants in response to treatment with SA or PAC + H_2_O_2_ under heat stress compared to plants sprayed with H_2_O, PAC, or SA + DMTU. Thus, there is no doubt that H_2_O_2_ at least plays a key role in mediating SA to enhance pollen viability under heat stress at the PMC meiosis stage of rice.

## Additional file

Additional file 1: **Figure S1.** Effects of SA on other three tapetum development genes in rice anther under heat stress. a, *DTC1* gene (Defective Tapetum Cell Death 1); b, *TDR* gene (Tapetum Degeneration Retardation); c, *Udt1* gene (Undeveloped Tapetum 1). Vertical bars denote the standard deviation (*n* = 3). (JPG 2513 kb)

## Abbreviations

APX: ascorbate peroxidase
CAT: catalase
DMTU: dimethylthiourea
*DTM1*: Defective Tapetum and Meiocytese 1
*EAT1*: Eternal Tapetum 1
*MIL2*: Microsporeless 2
NBT: nitroblue tetrazolium
PAC: paclobutrazol
PCD: programmed cell death
POD: peroxidase
ROS: reactive oxygen species
SA: salicylic acid
SOD: superoxide dismutase
TUNEL: terminal deoxynucleotidyl transferase-mediated dUTP nick-end labeling
