# Supplementary figures and images for: Salicylic acid reverses pollen abortion of rice caused by heat stress
Source: BMC Plant Biol. 2018 Oct 19;18:245. doi: 10.1186/s12870-018-1472-5 (PMC6194599; doi:10.1186/s12870-018-1472-5)

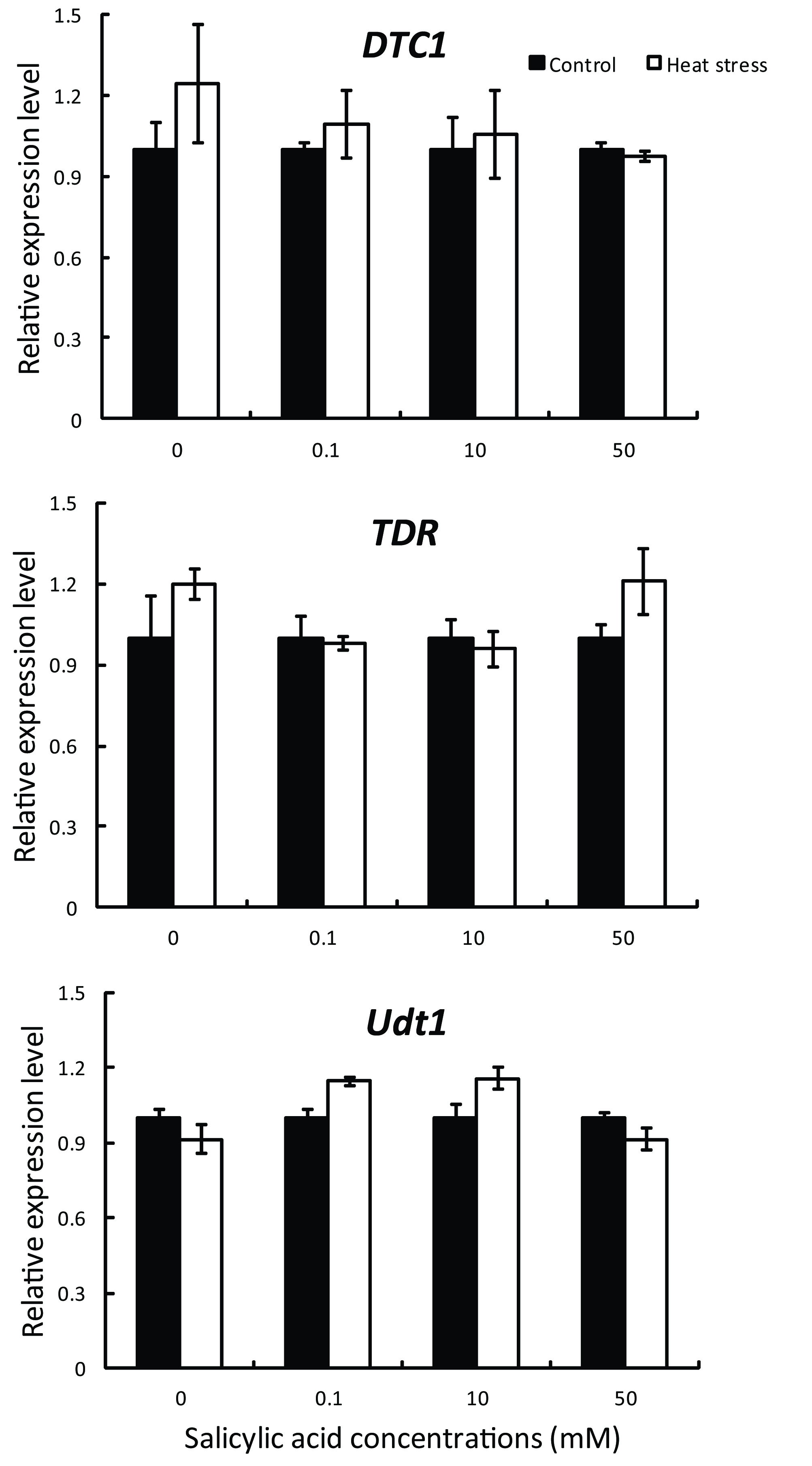

Supplement: Supplementary file 1 — Figure S1. Effects of SA on other three tapetum development genes in rice anther under heat stress. a, DTC1 gene (Defective Tapetum Cell Death 1); b, TDR gene (Tapetum Degeneration Retardation); c, Udt1 gene (Undeveloped Tapetum 1). Vertical bars denote the standard deviation (n = 3). (JPG 2513 kb) [file 12870_2018_1472_MOESM1_ESM.jpg]
